# Unveiling the Clinical Benefits of High-Volume Hemodiafiltration: Optimizing the Removal of Medium-Weight Uremic Toxins and Beyond

**DOI:** 10.3390/toxins15090531

**Published:** 2023-08-29

**Authors:** Cristian Pedreros-Rosales, Aquiles Jara, Eduardo Lorca, Sergio Mezzano, Roberto Pecoits-Filho, Patricia Herrera

**Affiliations:** 1 Departamento de Medicina Interna, Facultad de Medicina, Universidad de Concepción, Concepción 4070386, Chile; 2 Nephrology Service, Hospital Las Higueras, Talcahuano 4270918, Chile; 3 Departamento de Nefrología, Facultad de Medicina, Pontificia Universidad Católica de Chile, Santiago 8320000, Chile; 4 Departamento de Medicina Interna, Facultad de Medicina, Campus Oriente, Universidad de Chile, Santiago 7500922, Chile; 5 Instituto de Medicina, Facultad de Medicina, Universidad Austral, Valdivia 5110566, Chile; 6 Arbor Research Collaborative for Health, Ann Arbor, MI 48108, USA; 7 School of Medicine, Pontifícia Universidade Católica do Paraná, Curitiba 80215-901, Brazil; 8 Nephrology Service, Hospital del Salvador, Santiago 8320000, Chile

**Keywords:** hemodiafiltration, kidney failure, dialysis, uremic toxins, hemodynamic tolerance, cardiovascular disease, inflammation, oxidative stress, patient-reported outcomes, mortality

## Abstract

Dialysis treatment has improved the survival of patients with kidney failure. However, the hospitalization and mortality rates remain alarmingly high, primarily due to incomplete uremic toxin elimination. High-volume hemodiafiltration (HDF) has emerged as a promising approach that significantly improves patient outcomes by effectively eliminating medium and large uremic toxins, which explains its increasing adoption, particularly in Europe and Japan. Interest in this therapy has grown following the findings of the recently published CONVINCE study, as well as the need to understand the mechanisms behind the benefits. This comprehensive review aims to enhance the scientific understanding by explaining the underlying physiological mechanisms that contribute to the positive effects of HDF in terms of short-term benefits, like hemodynamic tolerance and cardiovascular disease. Additionally, it explores the rationale behind the medium-term clinical benefits, including phosphorus removal, the modulation of inflammation and oxidative stress, anemia management, immune response modulation, nutritional effects, the mitigation of bone disorders, neuropathy relief, and amyloidosis reduction. This review also analyzes the impact of HDF on patient-reported outcomes and mortality. Considering the importance of applying personalized uremic toxin removal strategies tailored to the unique needs of each patient, high-volume HDF appears to be the most effective treatment to date for patients with renal failure. This justifies the need to prioritize its application in clinical practice, initially focusing on the groups with the greatest potential benefits and subsequently extending its use to a larger number of patients.

## 1. Introduction

Dialysis has been shown to improve quality of life and increase survival in patients with kidney failure [1]. However, mortality in this group of patients remains unacceptably higher than other pathologies, including some forms of cancer [2], which is attributed to incomplete removal of some toxic uremic molecules, particularly those that are medium- and high-molecular-weight [3].

The introduction of membranes with substantial permeability and the combination of diffusive and convective transport have improved the removal efficiency of larger uremic toxins. Small molecules are effectively eliminated through diffusion, and low-flux hemodialysis (LF-HD) predominantly relies on this process to reinstate the internal environment, providing efficient clearance of small water-soluble molecules, such as creatinine and urea (molecular weight < 500 Da), but negligible removal of medium-weight uremic toxins. High-flux hemodialysis (HF-HD) uses membranes with a higher-molecular-weight cut-off and higher ultrafiltration coefficient (K_UF_), which translates into a higher hydraulic permeability. This allows the removal of higher-molecular-weight toxins through convection. This is made possible through filtration and backfiltration that is generated internally within the high-flux dialyzer [4]. The rationale for using hemodiafiltration (HDF) is based on simultaneous diffusive and convective transport, which combines the beneficial effects of diffusive HD with the advantages of large external convective volumes. Throughout HDF, the convective clearance for a given solute depends on the total ultrafiltered volume and the solute sieving coefficient of the membrane. According to the above, HDF has been defined by the EUropean DIALlysis (EUDIAL) Working Group as a blood purification therapy combining diffusive and convective solute transport using a high-flux membrane characterized by a K_UF_ greater than 20 mL/h/mmHg/m^2^ and a sieving coefficient for β2-microglobulin of greater than 0.6. Convective transport is achieved by an effective convection volume of at least 20% of the total blood volume processed [5]. This combination allows a greater removal of medium- and high-molecular-weight solutes by convection, maintaining the adequate removal of small solutes of the classical diffusive transport of conventional hemodialysis.

The use of external convection as a depurative method is known. Its use combined with diffusion to improve solute transport in chronic patients dates to 1978 when the term hemodiafiltration was first established [6]. The technological development of the technique allowed the production of on-line replacement fluid from an ultrafiltration process of the dialysis fluid, with low production cost and safety from a bacteriological point of view [7]. This innovation facilitated the extension of On-Line HDF (OL-HDF) and the increase in convective volumes, correlating linearly with increased β2-microglobulin (β2M) removal [8].

Although most of the clinical benefits can be attributed to the increased removal of medium and large uremic toxins, others seem to be explained by additional factors. These can be grouped into short-term, medium-term, and long-term benefits. Short-term benefits are those related to intradialytic or interdialysis effects, such as improved hemodynamic stability, reduced post-treatment fatigue, better weight management, and blood pressure control. Prolonged exposure to this treatment leads to intermediate benefits, including improved nutritional status and phosphorus levels, reduced inflammation and oxidative stress, alleviation of neurological symptoms and joint pain, enhanced response to erythropoietin, and better immune response [9]. 

While the short- and medium-term benefits are more relevant from the patient’s perspective and the concept of individualized and goal-oriented dialysis prescription [10], the sum of the benefits described above can translate into long-term benefits, such as improved survival [11].

Previous clinical experience suggested that there was no difference between HF-HD and low-volume HDF [12]. However, analysis of European data from the DOPPS study revealed that survival was significantly better in patients treated with HDF, but only in those with a “high efficiency” technique involving an infusion volume greater than 15 L [13]. These findings generated interest in the potential of HDF to improve clinical outcomes by increasing convective clearance, leading to the conduct of three clinical trials.

The CONTRAST study compared LF-HD with postdilution HDF and found no difference in overall mortality or cardiovascular mortality between the groups. However, in a secondary analysis, patients with a convection volume greater than 21.9 L/session were found to have significantly lower mortality rates (relative risk = 0.62; 95% CI, 0.41–0.83) [14]. The Turkish study compared HF-HD with postdilution HDF and found that the combined outcome of all-cause mortality or cardiovascular events showed no significant differences between the groups. As in the case of the CONTRAST study, subanalysis of the results showed that patients with a replacement volume greater than 17.4 L/session had a significantly lower mortality rate (relative risk = 0.71; 95% CI, 0.07–0.71; *p* = 0.01) [15]. The ESHOL study compared HF-HD versus postdilution HDF and found that the mean convective volume was 23.7 L/session and that patients assigned to the HDF group had a 30% decrease in the risk of all-cause mortality (95% CI, 0.53–0.92; *p* = 0.01) and a 33% decrease in the risk of cardiovascular death (95% CI, 0.44–1.02; *p* = 0.06). Compared to HD, the relative risk of death was 0.60 (95 % CI, 0.39–0.90) for convection volumes between 23.1 and 25.4 L and 0.55 (95 % CI, 0.34–0.84) for convection volumes greater than 25.4 L/session. In addition, a 55% reduction in infectious-cause mortality, a 61% decrease in the risk of death from a cerebrovascular event, and a 22% relative risk reduction in hospitalizations were observed [16].

The subsequent FRENCHIE clinical trial found no difference in mortality in its secondary analyses when comparing HF-HD with high-volume HDF, although in this case, convective volumes were not as high as in the ESHOL study [17]. In addition, studies collecting individual patient data from the last four clinical trials cited above show that the beneficial effect of HDF in reducing mortality is greater in patients who achieved larger convective volumes [18].

Despite the rapid acceptance of HDF, there is a lack of consensus within the nephrology community regarding its adoption in clinical practice. Furthermore, the skill set needed by the physicians to perform OL-HDF is slightly different from conventional HF-HD [19]. This leads to variability in its use in different regions and countries [20]. However, the recently conducted CONVINCE study across eight European countries addressed this issue and found that high-volume HDF is associated with a reduced risk of death compared to HF-HD. This study’s design effectively minimized confounding factors, supporting the significant survival advantage of high-volume HDF [21]. As a result, interest in this therapy has grown, driven by the need to comprehend its underlying mechanisms and the promising findings of the last evidence.

## 2. Impact of Hemodiafiltration on Biomarkers

The kidney can eliminate small molecules, as well as peptides with molecular weights between 10 and 30 kDa. In end-stage renal failure, dialysis has alleviated the classic symptoms of uremia by eliminating small molecules. However, it is possible to observe the persistence of “residual” signs and symptoms due to insufficient removal of larger molecules, resulting in physical and cognitive limitations, metabolic effects, and cellular dysfunction [22].

The conventional HD prescription allows the removal of approximately two thirds of the total body urea content during each treatment. Along with this, most of the small uremic toxins similar to urea, which can easily move via diffusion, are removed. The HEMO study showed that the results do not improve with an increase in fractional urea elimination above the current standard [23]. This is because the elimination of many solutes is limited due to their large molecular size, their binding to proteins, or their retention in body compartments. Therefore, plasma levels of these solutes remain much higher than normal urea levels in patients undergoing conventional dialysis [22].

Frequent complications, such as cardiovascular disease, anemia, bone disorders, and neuropathy, are still observed in patients with adequate Kt/V and have been correlated with the difficulty in removing uremic toxins in the molecular range of 5000 to 50,000 daltons [24].

The spectrum of medium-weight molecules includes β2M, used as an indicator of medium-toxin clearance and correlated as a risk factor for mortality; fibroblast growth factor 23 (FGF-23) along with parathyroid hormone (PTH), osteocalcin and osteoprotegerin, implicated in alterations of bone metabolism; indoxyl sulfate and p-cresol sulfate, which mainly bind to albumin and have been correlated with an increased risk of cardiovascular events; leptin, for its involvement in reducing appetite in dialysis patients; and free chains of immunoglobulins, hepcidin and homocysteine, which have also been identified as toxic inflammatory molecules in uremic patients [25].

Many studies of high-volume HDF have demonstrated superiority for the removal of these toxins compared with HF-HD [26,27], and the maximum benefit is achieved by exceeding 24 L of convective volume in a 4-h session, which is particularly important for β2M [8,28]. On the other hand, protein-bound toxins such as p-cresylglucuronide, hippuric acid, indole acetic acid, indoxyl sulfate, and p-cresol sulfate could be removed more efficiently by HDF [29].

### 2.1. Phosphatemia

**Figure 1:**
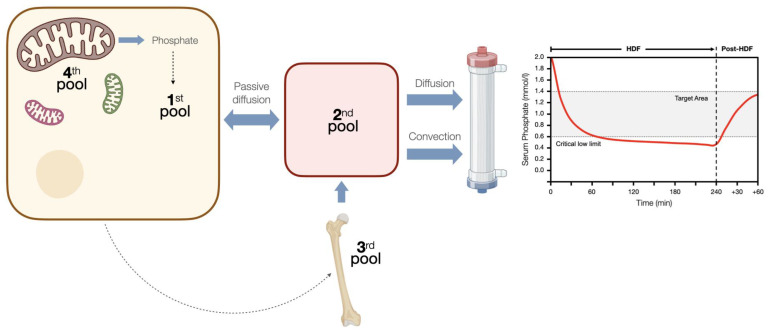
Phosphorus kinetics during hemodiafiltration. During hemodiafiltration, the levels of phosphate in the blood rapidly reach a plateau, below which they do not decrease. After therapy, there is a rebound effect that persists for over an hour. This can be explained by a four-compartment system with controlled kinetics, which responds to changes in the concentration of intracellular phosphate. Initially, there is a dynamic equilibrium established between the intracellular compartment (1st pool) and the extracellular compartment (2nd pool). Once the serum phosphate levels reach a critically low limit, the intravascular space receives additional phosphorus, potentially originating from a phosphate reservoir that has not yet been incorporated into the bone matrix (3rd pool). It is plausible that changes in the concentration of phosphate inside the cells trigger this control mechanism. Finally, when intracellular phosphate levels drop below a critical threshold (<0.97 mmol/L), there is an immediate release of phosphate from a fourth pool into the intracellular space. This 4th pool is believed to consist of phosphate derived from mitochondrial adenosine triphosphate (ATP). This mechanism serves to protect the intracellular environment from dangerously low concentrations of phosphate.

Studies with high-volume HDF are consistent in showing greater phosphorus removal compared to HD [30,31]. However, this could have a modest effect on predialysis phosphatemia, with an estimated drop of less than 15%, since patients who switch to HDF additionally improve their appetite and ingest greater amounts of protein and phosphorus [15,16,32], but also because phosphorus does not follow the same removal kinetics as urea, rapidly reaching a plateau phase after which phosphatemia levels do not fall any further [33,34], and a plasma rebound is observed after the end of the dialysis session, which also occurs in HDF [35]. This has been explained by kinetics of phosphorus body distribution that involve four compartments and a series of regulatory mechanisms that hinder the movement of phosphorus in an environment of abrupt changes in phosphatemia in a compensatory manner (Figure 1) [36]. Thus, it has been demonstrated with magnetic resonance spectroscopy that the phosphorus removed during dialysis mainly comes from the intracellular space and more specifically from the ATP deposits within the mitochondria [37]. These kinetics allow us to understand that the most efficient way to remove phosphorus is by prolonging the dialysis time [38,39] or through high-volume HDF for more than 4 h [34,40].

### 2.2. Oxidative Stress and Inflammation

Uremia is marked by heightened oxidative stress (OS) due to increased prooxidant molecules, poor oxidative product clearance, and deficient antioxidant defenses mechanisms [41]. This stress activates inflammatory agents, leading to damage of lipids, proteins, and DNA. Early chronic kidney disease stages show high OS, which worsens with declining kidney function and is notably higher in end-stage patients on HD compared to those on peritoneal dialysis [42]. Hemodialysis intensifies OS because the procedure activates prooxidant processes and depletes antioxidant molecules like vitamins and trace elements. Various factors contribute to this stress, including the interactions with non-compatible membranes and dialysate, anticoagulation, and the use of central venous catheters for vascular access and malfunctioning arteriovenous graft and fistulae [43].

Oxidative stress observed in uremia contributes to the inflammatory state of dialysis patients. Systemic inflammation is a multifactorial phenomenon but is partly attributed to the accumulation of uremic toxins that are difficult to remove (e.g., p-cresol and indoxyl sulfate) and bioincompatibility with the extracorporeal circuit, leading to complement activation and transcription of several proinflammatory cytokines, such as TNF, IL-1b, and IL-6 [44]. High levels of these cytokines are associated with lower survival in dialysis [45] and studies indicate that inflammation correlates strongly with atherosclerosis, with IL-6 being a good predictor of the inflammatory burden in this group of patients [46]. 

While some preliminary studies suggest that OL-HDF would modulate the expression of several genes involved in the pathogenesis of atherosclerosis, such as VEGF, PDGF, and IL-6, in peripheral mononuclear cells [47], other have shown that HDF would induce post-transcriptional modifications, decreasing the expression of genes involved in vascular damage. Switching from HD to OL-HDF has been shown to significantly reduce systemic inflammation and decrease the expression of pro-atherogenic miR-223 in endothelial vesicles, enhancing angiogenesis and reducing vascular calcification. The downregulation of miRNA-223 restores IGF-1 receptor gene expression, which prevents endothelial dysfunction and vascular calcification. This is the first study that attempts to explain the pathophysiology of the beneficial effect of OL-HDF on CV morbidity and mortality [48]. 

HDF has been shown to have a positive impact on oxidative stress compared to standard HD [49,50,51]. This could be attributed to the use of ultrapure dialysis fluid, hemodynamic improvement, better response to anemia treatment, and increased removal of medium- and large-weight molecules such as inflammatory mediators [43].

Preclinical studies and some prospective studies with small groups of patients have shown that convective transport of OL-HDF reduces cytokine levels and inflammatory parameters [52], as well as reducing the expression of proinflammatory CD14+ and CD16+ dendritic-cell-derived monocytes in patients undergoing HDF [53]. Along the same lines, a decrease in the inflammatory state has been observed in dialysis patients who switch to HDF, being more noticeable in diabetic patients. These results were explained by the downregulation of dendritic cell maturation and better control of the sympathetic nervous system by improving renalase levels in the group of patients submitted to HDF [54].

Therefore, although clinical trials aimed at finding specific benefits in this area are lacking, today, it is considered that high-volume HDF is the “least inflammatory” renal support technique, and it is possible that the decrease in chronic inflammation observed in these patients explains the decrease in chronic inflammation observed in these patients, which may explain the better results in long-term survival [44,55].

## 3. Impact of Hemodiafiltration on Clinical Outcomes

The unmet clinical needs of dialysis patients, such as residual uremic symptoms, high hospitalization rates, and high mortality, are not only due to the increasing age and comorbidities of the population, but also due to the intrinsic limitations of conventional HD, due to the inability of this dialysis modality to remove the full spectrum of uremic toxins [56]. The application of HDF has been shown to shorten the distance between dialysis and the native kidney. Thus, the impact on the removal of uremic toxins through convection correlates with good results in several clinical aspects not satisfied by dialysis mainly based on diffusive transport.

### 3.1. Anemia

Several observational and crossover studies have observed that patients on HDF have less anemia or have a better response to erythropoiesis-stimulating agents (ESAs), which could be the result of the significant removal of medium-sized proinflammatory molecules, including hepcidin, which also facilitates iron mobilization [57,58,59,60]. However, clinical trials show inconclusive results. While the CONTRAST study showed a decreasing trend in the use of ESA that did not reach a significant difference [14], the Turkish OL-HDF study showed a significantly lower weekly erythropoietin dose in patients undergoing HDF [15]. The ESHOL study observed no significant differences in hemoglobin levels, transferrin saturation index, or ferritin levels and the doses of ESA were similar between patients on high-volume HDF and those in the HF-HD group [16]. In the absence of conclusive evidence, it is possible to state that by the various mechanisms mentioned, particularly the inflammatory environment improvement, HDF seems to reduce the dose of ESA [61,62], and this would also translate into a reduction in the pharmacological burden and associated costs [63].

### 3.2. Immune Response and Infections

Patients with kidney failure have a significantly higher risk of infectious complications, being the first cause of hospitalizations and the second cause of death in dialysis after cardiovascular causes; thus, it is possible that both pathogenic mechanisms are linked to the immune disorders of chronic kidney disease [64].

Thus, it has been shown that several medium and large uremic toxins impair the immune system. For example, free light chains of immunoglobulins, retinol-binding protein-4 (RBP-4), fibroblast growth factor-23 (FGF-23), and alpha-1 glycoprotein reduce leukocyte activity by different mechanisms [65]. Similarly, degranulation-inhibiting protein (DIP) and granulocyte inhibitory protein (GIP) inhibit in vitro glucose uptake and polymorphonuclear leukocyte chemotaxis, while complement factor D decreases immunocomplex clearance and inhibits granulocyte degranulation [66]. All these toxic molecules are potentially removed by high-volume HDF [67] and the ESHOL clinical trial confirmed this hypothesis by demonstrating that patients treated with high-volume HDF had a 55% reduction in infectious mortality compared to patients undergoing HF-HD, along with a 22% reduction in the rate of hospitalizations for the HDF group [16]. Although the main mechanism of benefit appears to be the improved clearance of GIP, the improved hemodynamic stability observed with HDF may play an important role in reducing episodes of intestinal ischemia and subsequent bacterial translocation [68].

Although there is no definitive evidence on the effects on the immune response, sustained seroprotection and increased lymphocyte proliferation have been observed in response to vaccination against Influenza A in chronic renal patients undergoing HDF compared to those treated with HD [69]. While no other studies confirm this effect with other vaccines, such as hepatitis B and anti-pneumococcal, there is recent evidence that HDF patients vaccinated against SARS-CoV-2 have higher antibody levels and a sustained response over time compared to those on HD [70,71].

### 3.3. Nutritional Effects

Dialysis patients have a high prevalence of malnutrition inflammation complex syndrome (MICS). This condition is associated with significantly elevated rates of hospitalization and mortality rates in this group of patients [72].

The anorexia and protein-energy wasting observed in dialysis patients have been extensively linked to the accumulation of medium molecules and increased levels of adipokine and leptin, IL-6, TNF-α, and IL-1b, which has been associated with lower albumin and pre-albumin levels, an inverse correlation with muscle mass, and lower levels of physical endurance [65]. 

Leptin (16 kDa), which is usually elevated in dialysis patients and known for its anorexigenic effect [73], is more effectively removed with HDF [74], which may contribute to increased appetite. A prospective randomized controlled trial of 33 adult patients reported that those treated with HF-HD had a reduction in lean tissue and body cell mass compared with patients treated with HDF after a 12-month follow-up and with a higher estimated protein intake in patients undergoing HDF [75]. 

The scenario in which HDF has the greatest nutritional impact is in children since the patients’ growth is used as a parameter of adequate nutrition and effectiveness in the dialysis dose. Despite the daily administration of recombinant human growth hormone (GnRH), childhood growth retardation remains a frequent problem in children undergoing chronic dialysis [76]. Initial studies showed that intensive HDF (6 times per week) promotes a positive effect on growth, even greater than using GnRH alone [77]. The HDF, Heart, and Height (3H) study was the first prospective longitudinal multicenter study comparing the results of OL-HDF results with conventional HD in children and included 28 pediatric dialysis centers in 10 countries [78]. Although it is not a randomized controlled trial (difficult to perform due to the small number of children on dialysis and high rate of transplantation), the 3H study included almost 40% of children on dialysis in Europe among incident and prevalent patients, confirming a series of benefits of the technique in the pediatric population, such as improvement in cardiovascular goals, less inflammation, better control of bone mineral disease, and better quality of life. Noteworthy was the improvement in the growth curve in the HDF group compared to those on HD, which was independent of GnRH. Furthermore, an inverse correlation was observed between the growth curve and β2M levels, suggesting that removal of medium-molecular-weight molecules, including inflammatory cytokines and endogenous inhibitors of somatomedin (IGF-1) and gonadotropin, may partly alleviate growth hormone resistance in pediatric dialysis patients [79].

Since convection is the major determinant of low-molecular-weight protein loss and uremic protein removal, this is an HDF target per se [80]. Amino acid and albumin loss can be expected to be more pronounced with increasing transmembrane pressure compared to HD [81]. In fact, it has been reported that there is a moderate loss of albumin in the effluent, especially when high convective volumes are used [82]. The amount of albumin loss would be lower with usual convective goals (<30 L), ranging from 2 to 3 g per session, which would be similar to the loss observed with high-flux filters in conventional HD and peritoneal dialysis, which would not determine a risk of malnutrition [83,84,85]. However, it should be considered that some studies have observed significant losses of amino acids and albumin when higher permeability filters are used, sterilized with steam or in cases in which very high convective volumes are used, for example, in the predilution mode [86,87,88]. For this reason, the dialyzer selection, modality, and prescription must be careful, particularly in those patients who present hypoalbuminemia, a risk of malnutrition, and in whom sufficient protein intake cannot be assured.

### 3.4. Effects on Cardiovascular System

One way of measuring cardiovascular risk is through carotid intima-media thickness (cIMT) measured by ultrasound, which correlates with arterial stiffness and cardiovascular events [89,90]. In the 3H study, it was observed that the pediatric patients in HDF had a significantly lower cIMT score than those who remained on HD. The predictors of higher cIMT were greater interdialysis weight gain, higher systolic pressure, and higher β2M levels [78]. The development of arterial stiffness has been related to chronic inflammation, oxidative stress, and bone mineral disease characteristic of kidney failure. Thus, the potential benefits of HDF in this scenario could be the improvement in endothelial function and the decrease in vascular calcifications by reducing oxidative stress [48].

Although some studies suggest that endothelial function and arterial stiffness improve with the use of HDF [91], so far, it has not demonstrated a positive impact over HD in the alterations of intratherapeutic cardiac motility [92], in arterial stiffness evaluated by pulse wave velocity [93], or in the improvement in left ventricular mass and ejection fraction [94].

Another aspect that could influence cardiovascular outcomes is hydrosaline overload. Although the volemia status mainly depends on interdialysis intake and residual renal function, the large amount of replacement fluid provided during OL-HDF could generate a positive sodium balance and fluid accumulation. Initial studies observed higher predialysis natremia using HDF, suggesting a lower sodium elimination during this technique, which would be partly responsible for the better cardiovascular stability during therapy [57,95,96]. However, later studies ruled out an association between high convective volumes and hydrosaline overload [97].

Furthermore, the preservation of residual renal function has been associated with a series of benefits, including lower plasma levels of uremic toxins, less inflammation, better nutritional status, better water balance, and increased survival, even with urea clearance <3 mL/min [98]. It has been observed that residual renal function is preserved more in peritoneal dialysis than in HD, which has been attributed to the greater frequency of hypotension episodes and periods of dehydration in HD patients [99]. On the other hand, the use of ultrapure water and more biocompatible membranes would make it possible to preserve renal function as efficiently as peritoneal dialysis [100,101]. Theoretically, by having a better hemodynamic profile and decreasing the microinflammatory environment, OL-HDF could be more useful than HD in maintaining urine volume over a prolonged period [102]. However, no extensive observational studies or clinical trials have presented results in this area [103].

### 3.5. Hemodynamic Stability

The poor tolerance observed in dialysis mainly refers to episodes of clinical and subclinical hypotension within therapy, which are associated with the hypoperfusion of vital organs, such as the brain, intestine, heart, and kidneys, as well as increased mortality [104]. The prevalence of this complication varies according to the definition used [105]. The most common working definition falls in SBP ≥ 20 mmHg or MAP ≥ 10 mmHg associated with symptoms and requiring interventions such as reduced ultrafiltration or administration of intravenous fluids, or both [106].

Risk factors associated with hemodynamic intolerance on HD include diabetes mellitus; cardiovascular disease with systolic and diastolic dysfunction, ischemic heart disease, arrhythmias, and severe vascular calcification; autonomic dysfunction; poor nutritional status; hypoalbuminemia; female sex; age > 65 years; predialysis SBP < 100 mmHg; high body mass index; and severe anemia [107,108].

Many observational studies indicate that the use of HDF improves hemodynamic tolerance by decreasing episodes of symptomatic hypotension. These findings have been confirmed by multiple controlled trials [16,17,96,109]. The mechanisms behind intradialytic hypotension are multiple and HDF seems to act on several of them directly and indirectly [110,111]. 

**Figure 2:**
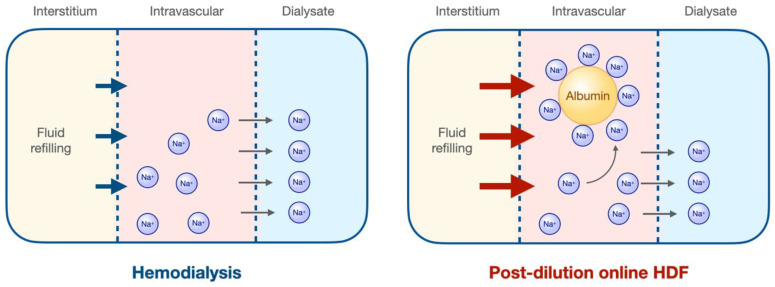
Gibbs–Donnan effect in hemodiafiltration. During conventional hemodialysis, sodium is removed by convection and diffusion until equilibrium is reached, without causing significant changes in osmotic pressure. However, in postdilutional hemodiafiltration, significant intermittent ultrafiltration occurs, resulting in secondary hemoconcentration, which causes an increase in the albumin concentration on the blood side of the membrane. When large negatively charged particles, such as albumin, cannot diffuse across a semipermeable membrane and are present in a fluid compartment such as the vascular compartment, they attract positively charged ions, especially sodium, which is the most abundant in plasma. This generates an osmotic gradient that favors the movement of fluids into the blood compartment. At Gibbs–Donnan equilibrium, this osmotic pressure reaches approximately 6–7 mmHg, which could facilitate fluid refilling from the interstitium and provide hemodynamic stability.

One of the mechanisms may be explained by the sodium distribution when high convective volumes are applied. During a dialysis session, water and sodium are mainly removed by ultrafiltration and a small part also by the negative sodium gradient between dialysate and plasma, until a diffusive equilibrium is reached. During an OL-HDF session, where large convective volumes are removed, albumin, behaving as a non-removable anion, concentrates on the blood side of the dialysis membrane, which favors the retention of sodium, which binds to albumin to establish an electrical equilibrium, explained by the Gibbs–Donnan effect [110,112]. Increased sodium retention will increase osmolarity in the blood compartment, facilitating fluid refilling from the interstitial compartment (Figure 2) [113]. The better hemodynamic tolerance could also be explained by a possible increase in pre-dialysis systolic blood pressure, as observed in some studies [57,95,96,114]. Although this possible beneficial effect could lead to hydrosaline overload, no persistent increase in natremia has been observed in clinical trials [17], and no markers of fluid overload are present with the use of high-volume HDF. Moreover, based on the improved hemodynamic tolerance observed, patients with persistent hypotension and the inability to achieve the indicated dialysis time can be transferred to HDF, after which they can achieve the desired ultrafiltration rates [106,107]. On the other hand, studies aimed at evaluating total sodium mass have ruled out a positive balance in patients undergoing HDF [115,116,117]. 

Although more specific studies are needed to correlate the Gibbs–Donnan effect and its clinical impact in HDF patients, it is most likely that the hemodynamic benefits observed depend on several factors and not on this specific mechanism.

Following the observations that low dialysate temperature provided better hemodynamic tolerance [118], it was postulated that the lower frequency of hypotension during OL-HDF sessions was due to blood cooling as the main protective factor. Since energy loss was greater in the extracorporeal system, even with the same programming as in HD, the blood returning to the patient was cooler during OL-HDF than during conventional HD. However, when comparing OL-HDF with conventional HD at lower temperatures and strict temperature control, a comparable occurrence of symptomatic hypotension to OL-HDF has been noted [119,120]. Despite the above, multiple observational studies and controlled trials have shown better hemodynamic tolerance in high-volume HDF, as it probably results in a lower core temperature and a negative heat balance due to the use of larger convective volumes [121,122].

**Figure 3:**
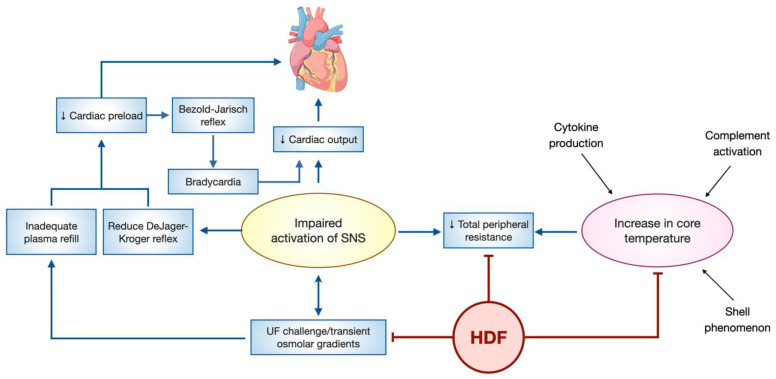
Role of hemodiafiltration in intradialytic hypotension. During hemodialysis and ultrafiltration (UF), normal compensatory responses involve activation of the sympathetic nervous system and the renin–angiotensin–aldosterone system, in addition to adequate plasma refilling. All this facilitates blood pressure maintenance through increased venous return and cardiac preload, increased cardiac output, and arteriolar vasoconstriction. When any of these compensatory mechanisms are impaired, hypotension is observed. Hemodialysis leads to an increase in core body temperature, which has been attributed to the blood–membrane interaction that generates complement activation and cytokine release. In addition, reduced heat loss through the skin due to peripheral vasoconstriction in response to UF may increase body temperature (shell phenomenon). As a consequence of this heat accumulation, loss of vascular tone may occur, resulting in hypotension. Hemodiafiltration (HDF) can improve hemodynamic stability by acting on several of these mechanisms.

Contrary to expectations, isothermal HD leads to an increase in core temperature. The underlying pathophysiology of this phenomenon is complex, involving multiple mechanisms, including cytokine production triggered by the bioincompatibility of the extracorporeal circuit, complement activation when biocompatible membranes are utilized, and alterations in the core temperature set point [123]. Additionally, initial peripheral vasoconstriction in response to hypovolemia plays a role, resulting in a "shell phenomenon" characterized by an initial heat accumulation causing a rise in core temperature, followed by subsequent vasodilation [124]. This leads to a decrease in peripheral vascular resistance, which is more marked in those patients with alterations in the activation of the sympathetic nervous system and decreased DeJager–Kroger reflex, as observed in elderly patients with dysautonomia, diabetes, or advanced amyloidosis, in whom the fall in preload ends up generating sudden episodes of intra-dialysis hypotension [125]. In such cases, hypothermic dialysis has shown potential benefits by partially improving the mechanism of intra-dialysis hypotension. Theoretically, it is possible that HDF may contribute to the decreased core temperature by removing inflammatory molecules (Figure 3).

A relevant cause traditionally associated with intradialytic hypotension is the inflammatory reactions triggered by exposure to the extracorporeal circuit [126]. While advancements in membrane compatibility and the use of ultrapure water have reduced its significance, the inflammatory impact and elevation of inflammatory molecules during HD remain well recognized [65]. Numerous studies have indicated that HDF leads to decreased levels of IL-6 and ultrasensitive C-reactive protein compared to conventional HD [26,52,127]. However, a direct correlation between the reduction in inflammatory markers and improved hemodynamic tolerance has yet to be established.

Furthermore, it has been suggested that other indirect mechanisms contribute to enhanced peri-dialytic hemodynamic stability, including the improved preservation of residual diuresis, better management of anemia, and positive effects on nutritional status and physical activity [128].

### 3.6. Amyloidosis and Joint Pain

The introduction of high-flux membranes and improvements in the quality of HD water, particularly microbiological aspects, have effectively reduced the inflammatory environment and plasma levels of β2M, the primary molecule implicated in dialysis-related amyloidosis (DRA) [129]. Nevertheless, clinical manifestations of amyloidosis are still observed in certain patients, particularly those who initiated dialysis more than ten years ago [130]. Renal transplantation is considered the optimal treatment for established DRA and should be prioritized [131]. However, donors’ limited availability and the presence of various acknowledged barriers frequently hinder their eligibility for inclusion in transplant programs [132].

Considering that the clearance of β2M exhibits a linear relationship with convective volume, it can be anticipated that high-volume HDF offers the most effective means of removing β2M [8,40]. This approach may be recommended for patients with β2M levels ≥27 mg/L in order to reduce the risk of mortality. It is also recommended for those with symptomatic manifestations of amyloidosis that significantly affect their quality of life, and cases such as arthropathy, bone cysts with pathologic fractures, carpal tunnel syndrome, systemic involvement, and symptomatic autonomic dysfunction [133,134].

Elevated levels of β2M alone do not sufficiently explain amyloid deposition, as demonstrated by experimental studies [135]. The pathogenesis of DRA involves an increase in molecules that modify the conformation of β2M, facilitating the stabilization of amyloid fibrils. This process is favored by the inflammatory environment associated with uremia [130,136]. Therefore, the additional benefit of HDF, compared to high-flux HD, lies in the significant reduction in β2M levels in conjunction with the attenuation of the inflammatory milieu, contributing to a lower incidence of DRA.

It is worth noting reports describing improvements in osteoarticular symptoms related to enhanced clearance of medium and large uremic toxins. Patients who transition from HD to high-volume HDF have experienced an increased range of motion in the extremities and reduced joint pain [137,138,139].

### 3.7. Neurological Symptoms

Neurological complications significantly contribute to physical disability in dialysis patients. Despite advancements in dialysis techniques, neuropathy affects over 70% of patients undergoing dialysis for more than ten years. This neuropathy is primarily attributed to the accumulation of medium-weight uremic toxins, leading to segmental demyelination and axonal degeneration [140]. Clinical manifestations include insomnia, irritability, restless legs, and pruritus, with peripheral polyneuropathy being the most prevalent long-term disorder. It presents as sensory loss, paresthesia, reduced tendon reflexes, muscle atrophy, and weakness [141].

Preliminary reports suggest that HDF may have a preventive or slowing effect on the progression of peripheral neuropathy. Observations indicate that nerve excitability remains close to normal when HDF is used in incident patients, likely due to the early elimination of medium-weight uremic molecules [142,143]. Switching prevalent dialysis patients to HDF has also been associated with a significant reduction in uremic pruritus and restless legs [144,145,146,147].

However, it is relevant that while these symptomatic improvements and potential quality-of-life benefits are reported, uremia-related neurological damage leading to sensorimotor polyneuropathy has not shown significant treatability. In the FINESSE clinical trial, which spanned four years and included 124 patients randomized to HF-HD or high-volume HDF, the use of HDF resulted in a higher clearance of β2M, but no notable differences in the progression of neuropathy were observed between the two groups [148].

### 3.8. Patient-Reported Outcomes

Morbidity and mortality outcomes should not be the sole determinants for therapeutic interventions. Patient-reported outcomes, such as health-related quality of life (HRQoL) and its impact on decision making, are increasingly recognized as equally important. Fatigue is considered a central element in assessing the effects of dialysis on HRQoL [10]. However, the evidence on the effects of HDF on quality of life remains insufficient, with discrepancies in the results due to the focus of studies on classical clinical outcomes [149].

While some studies suggest no differences in HRQoL scores between HD and HDF patients [149,150,151,152], others have demonstrated beneficial effects on various aspects of quality of life. These include improvements in the social, physical, and professional domains, characterized by reduced fatigue, joint pain, stiffness, cramp frequency, pruritus, and episodes of hypotension [153,154]. However, current evidence does not support the significant improvement in cognitive function or in the physical and mental health domains with HDF [151,155]. The HDFit trial showed a mild improvement in physical activity, as reflected by higher step counts in patients treated with HDF [152]. However, HDF did not significantly affect sleep duration in the same study [156].

One challenge in analyzing these results is the limited sample sizes and short follow-up periods in most studies. Additionally, generic HRQoL questionnaires, such as SF-36 or EQ-5D, may not adequately capture the effects of dialysis on patient perception, while specific questionnaires like the Kidney Disease Quality of Life (KDQOL) survey have validity concerns [157].

Thus far, two clinical trials have aimed to evaluate general and cardiovascular mortality, as well as benefits in hospitalizations, quality of life, and cost effectiveness. The first study already conducted is the CONVINCE prospective randomized controlled trial (high convective volume versus high-flux HD) [158]; the second is the ongoing multicenter study H4RT (high-volume HDF versus high-flux HD registry trial) [159]. The H4RT study uses a conventional registry system to assess HRQoL, whereas the CONVINCE study addresses a much larger spectrum of quality-of-life domains, based on the core elements accepted by the SONG initiative (Standardised Outcomes in Nephrology initiative, International Consortium for Health Outcomes Measurement) [10]. For this, the CONVINCE study used an electronic registry system called PROMIS^®^ (Patient Reported Outcomes Measurement Information System). This system has an innovative approach to the development and evaluation of patient reports, adapting the questions individually from large data banks, which makes it a more efficient, flexible, and accurate tool [160,161].

## 4. Impact of Hemodiafiltration on Long-Term Outcomes

Based on data from the DOPPS (Dialysis Outcomes and Practice Patterns Study) [13], which demonstrated significantly longer survival in patients treated with HDF using an infusion volume of >15 L, multiple clinical trials have subsequently confirmed the survival benefit associated with high convection volumes [14,15,16].

A 2015 Cochrane review acknowledged the potential reduction in cardiovascular mortality with HDF but found insufficient evidence to support a decrease in all-cause mortality [149]. However, the CONTRAST and Turkish clinical trials showed benefits in survival when high convection volumes were achieved. For its part, the ESHOL study showed a clear decrease in all-cause mortality, even after reanalyzing its intention-to-treat data without censures and when all-cause mortality was considered with time-dependent and competing risks for transplantation [162]. In addition, several databases from different countries and a pooled individualized data analysis consistently indicate a survival benefit associated with a higher convective dose [18,163,164,165,166,167,168].

It is crucial to consider that previous studies employed different dialysis techniques (low-flux and high-flux), and there is a heterogeneity in vascular access, blood flow, treatment times, and achieved convection volumes. Furthermore, confounding factors may arise as patients achieving higher convection volumes tend to have fewer comorbidities, potentially influencing the interpretation of the survival benefit [169]. These discrepancies suggest that the positive effects of higher convection volumes cannot be universally generalized to the overall population of dialysis patients. Consequently, guidelines differ in their recommendations, as high-volume HDF is not uniformly considered a definitive technique until rigorously designed randomized controlled trials are conducted to evaluate potential confounding variables.

Based on this rationale, two large clinical trials have been designed. The multicenter H4RT study aims to include more than 30 centers in the United Kingdom, with a follow-up duration ranging from 23 to 50 months, and it is still ongoing, with patient recruitment expected to be completed in 2024 and the first results reported in 2025 [159]. On the other hand, the CONVINCE study recently published its primary outcome results, involving the participation of 61 centers across eight countries. With a median follow-up period of 30 months, a total of 1360 patients were randomized, including 683 patients receiving HF-HD with Kt/V > 1.4, and 677 prevalent patients undergoing postdilutional HDF, achieving a minimum volume of ≥23 L per session. A total of 118 patients (17.3%) in the HDF group died from any cause, equivalent to 7.13 events per 100 patient-years. In comparison, 148 patients (21.9%) in the HD group died from any cause, equivalent to 9.19 events per 100 patient-years. The hazard ratio was found to be 0.77 (with a 95% confidence interval of 0.65–0.93 and a *p*-value of 0.005), indicating a lower risk of death in the HDF group compared to the HD group [21].

This trial was conducted in a pragmatic manner, where all data were collected during routine clinical practice. Additionally, it involved patients who were eligible for HDF and consistently received high doses throughout the follow-up period, regardless of the type of vascular access. As this study was randomized and controlled, the observed findings are unlikely to be influenced by confounding factors related to patient selection. Thus, the results of the CONVINCE study strongly support the conclusion that high-volume HDF can provide a significant survival benefit with considerable clinical relevance. A summary of the clinical trials comparing the mortality of patients on HDF and HD can be found in Table 1.

## 5. Future Directions

Although there is a clear recognition that uremic toxicity has a significant impact on dialysis outcomes, influencing longevity and affecting patient well-being, much less is known about the impact of strategies to improve uremic solute removal and its impact on improving outcomes. High-volume HDF has shown promising results in improving patient outcomes, which is likely, and at least partially, related to uremic toxin removal. However, there are several important questions and challenges that need to be addressed to advance this technique and ensure its widespread adoption.

One key area for improvement is the complete removal of uremic toxins that are not effectively eliminated with the current technique, particularly some protein-bound toxins. Future research should focus on identifying these specific molecules, developing advanced membrane materials, exploring innovative filtration strategies, and investigating hybrid therapies or adsorption techniques to enhance toxin removal and optimize patient outcomes.

Another crucial aspect is determining the appropriate convection volume for different patient populations. Standardized protocols should be developed to assess individual patient characteristics such as body size, residual renal function, comorbidities, and toxin levels. This personalized approach will guide the selection of the optimal convection volumes, ensuring effective toxin clearance while minimizing the risk of complications associated with excessive convection.

Furthermore, the spread of high-volume HDF to a global scale is essential. Currently, the technique is primarily used in Europe and Japan. Overcoming barriers to dissemination requires raising awareness among healthcare providers, addressing the affordability and accessibility of equipment and resources, and bridging infrastructure and training gaps. International collaborations, knowledge-sharing platforms, and advocacy efforts will play pivotal roles in promoting the adoption and implementation of high-volume HDF worldwide.

## 6. Conclusions

Compared to high-flux hemodialysis, high-volume HDF appears to be the most advanced approach to prolonging the survival of patients with renal failure while awaiting renal transplantation. Therefore, it should be considered the preferred technique in this clinical context without specific indications for individual patients, although benefits in specific subgroups still need to be explored. While the long-term benefits seem to be more evident in younger patients with a better nutritional status and lower cardiovascular burden [170], short-term benefits and symptom relief may be more relevant in groups with higher comorbidity [171].

Advancements in technology have made high-volume HDF safe and accessible, with over two decades of research backing its implementation. It will be important to advance the understanding of the causal role of the effective elimination of larger uremic toxins, which significantly influences biomarkers in the improved biochemical, clinical, and patient-reported outcomes observed when HDF is compared to standard HD.

Going forward, HDF depends on further advances in our understanding of uremic toxin elimination and the optimization of convection volumes tailored to individual patients. It is essential to continue conducting ongoing research, fostering collaboration among stakeholders, and advocating for the expanded utilization of HDF globally. By addressing these challenges, high-volume HDF has the potential to become a transformative therapy for patients with kidney failure worldwide.

## Figures and Tables

**Table 1:** Randomized controlled trials comparing patient mortality between hemodiafiltration and hemodialysis.

Study Parameter	CONTRAST Study [14]	Turkish Study [15]	ESHOL Study [16]	FRENCHIE Study [17]	CONVINCE Study [21]
Comparison	LF-HD (*n* = 358) versus HDF (*n* = 356	HF-HD (*n* = 391) versus HDF (*n* = 391)	HF-HD (*n* = 450) versus HDF (*n* = 456)	HF-HD (*n* = 191) versus HDF (*n* = 190)	HF-HD (*n* = 677) versus HDF (*n* = 683)
Mean follow-up (years)	3.04	1.89	1.91	1.64	2.5
Delivered convection volume	20.7 L per session	>17.2 L per session + net UF	22.9–23.9 L per session	21 L per session	24.8–25.7 L per session
Primary outcome	All-cause mortality	All-cause mortality + nonfatal CV events	All-cause mortality	Intradialytic tolerance	All-cause mortality
HR for primary outcome (95% CI)	0.95 [0.75–1.20]	0.82 [0.59–1.16]	0.70 [0.53–0.92]	0.94 [0.51–1.76]	0.77 [0.65–0.93]
Secondary outcomes (HR, 95% CI)	Fatal + nonfatal CV events (1.07, 0.83–1.39)	CV and overall mortality (0.72, 0.45–1.13)	CV mortality (0.67, 0.44–1.02); infection-related mortality (0.45, 0.21–0.96)	All-cause mortality (0.83 0.52–1.33)	CV mortality (0.81, 0.49–1.33); infection-related mortality (0.69, 0.49–0.96)
Potential limitations	Target volume was not achieved in 50–66% patients. The control group used low-flux HD. Mortality significance was observed only with post hoc analysis.	Significance in mortality reduction only observed with post hoc analysis. Underpowered (lower-than-anticipated event rate).	HDF group was younger, less diabetic, and had a lower Charlson Comorbidity Index (CCI). RRF not monitored.	This study was underpowered (recruitment target not met). Mortality for entire kidney failure population was low.	The sample size was smaller than calculated, and it is difficult to interpret data related to cardiovascular mortality and hospitalizations due to the COVID-19 pandemic.

CONTRAST, Convective Transport; ESHOL, Estudio de Supervivencia de Hemodiafiltración On-Line; FRENCHIE, French Convective versus Hemodialysis in Elderly; CONVINCE, Comparison of High-dose HDF with High-flux HD; LF-HD, low-flux hemodialysis; HDF, hemodiafiltration; HF-HD, high-flux hemodialysis; UF, ultrafiltration; HR, hazard ratio; 95% CI, 95% confidence interval; HD, hemodialysis; CV, cardiovascular.

## Data Availability

Not applicable.
